# The Economic Impact of Cutaneous Leishmaniasis in Sri Lanka

**DOI:** 10.1155/2018/3025185

**Published:** 2018-10-11

**Authors:** Tharaka Wijerathna, Nayana Gunathilaka, Kithsiri Gunawardena

**Affiliations:** Department of Parasitology, Faculty of Medicine, University of Kelaniya, Ragama, Sri Lanka

## Abstract

Cutaneous leishmaniasis (CL) is a neglected tropical disease which affects mainly the poorest communities in developing countries. Considering the limited published information on economic impact of CL in Sri Lanka, the current study was conducted with the objective of revealing the nature and magnitude of the economic impact of CL in three selected disease endemic regions in Kurunegala District, Sri Lanka. The patient records of CL notified of relevant Medical Officer of Health (MOH) office during 2013- 2016 were obtained. Patient households were visited and data collection was done using an interviewed administered questionnaire. The majority of patients (57%) were economically active at the time of infection. Of them, 65% were the only contributors to household economy. Total median costs including both direct and indirect costs were 66.85 USD (Rs. 10,831) (IQR = 57.26 - 86.78 USD), while total median economic loss to households was 61.27 USD (Rs. 9,927) (IQR= 49.61- 75.04 USD). From provider perspective, total median cost per patient was 22.83 USD (Rs. 3,696). The mean total economic loss was denoted as 65.26 USD (Rs. 10,572) which is about 5.4% of the annual household income and 20.9% of the mean annual per capita income of the study population. Although economic impact of CL infection is not catastrophic according to current interpretation, the infection may have significant economic impacts on households when considering the mean economic loss to household as a percentage of the mean annual per capita income of the population.

## 1. Introduction

Leishmaniasis is a complex vector-borne disease caused by* Leishmania *sp. (Kinetoplastida: Trypanosomatidae) which transmits by infected female sand flies (Diptera: Psychodidae). The disease is endemic in 98 countries with more than 278,000 cases per year [1]. It is considered to be a disease of poverty affecting the poorest of poor [2, 3]. Households with low incomes who are living in precarious conditions such as mud or grass covered houses are at a higher risk for acquiring infection [4]. In general, outdoor occupational exposure is considered an established risk factor as occupational groups such as those who work in close proximity to forest areas are at greater risk [5].

There are three main forms of leishmaniasis, namely, visceral leishmaniasis (VL), cutaneous leishmaniasis (CL), and mucocutaneous leishmaniasis (MCL). Of these forms, the VL is considered to be the most virulent disease orientation. In Sri Lanka, CL is the predominant form of disease [6]. More than 2,000 cases have been identified from 2000 to 2009 [7]. Nearly 8,487 patients have been recorded during 2009-2016 representing at least one case from all 25 administrative districts. Although leishmaniasis patients have been reported from all districts, Anuradhapura, Hambantota, Polonnaruwa, Kurunegala, and Matara were the districts identified as endemic areas with more than 100 new patients annually. It is interesting to highlight that the number has been increased nearly 18-fold in the District of Kurunegala when compared to the cases reported in 2009 [8].

Sri Lanka is the newest reported focus of leishmaniasis in the Indian subcontinent where the disease is caused by the most virulent visceralizing species,* L. donovani*. The potential for visceralization in the cutaneous variant of* L. donovani* in Sri Lanka is not known until recently [9]. However, some recent studies have indicated that there is no visceralizing potential in* L. donovani *strain found from Sri Lanka [10].

Public health importance of the leishmaniasis disease in Sri Lanka is undervalued due to limited knowledge on disease burden, including socioeconomic aspects of the disease. The studies assessing financial and economic burden of leishmaniasis may be useful to understand the economic impact and financial consequences due to leishmaniasis incidence to households in some countries in the Indian subcontinent [4, 11, 12]. However, such aspects are inadequately evaluated in Sri Lanka.

Other tropical neglected diseases, in particular the helminthic infections, have been successful in gathering international attention and funding by showing the low cost of control interventions and their cost effectiveness. However, to date, only limited studies have addressed the costs of leishmaniasis illness, either from the household or from the health care provider perspective, in Sri Lanka. Therefore, information on direct and indirect cost associated with disease incidence would allow decision making authorities and policy planners to increase investment, facilities, and resource allocation in control policies and programs. Hence, the purpose of the present study was to determine the economic burden of CL on households in three selected disease high risk areas in Kurunegala District, Sri Lanka.

## 2. Methods

### 2.1. Study Area

Kurunegala District (7°45′N, 80°15′E) belongs to North Western Province of Sri Lanka covering 4812.7 km^2^. Its population is nearly 1, 676,000 with 439,065 households. The majority (50%) of them are inhabitants of rural areas (163, 6743) followed by urban areas (31,421) and estate (7,836) [13]. About 32.6% of the population is involved in agricultural practices as their main source of income while 36.6% is involved in other nonagricultural services and the rest depend on industries [14]. Kurunegala is one of the districts with high cutaneous leishmaniasis (CL) prevalence. According to the patient records from 2009 to 2016, there is nearly 18-fold increase in the leishmaniasis incidence indicating the highest increase rate of the disease occurrence.

### 2.2. Selection of Study Sites

**Figure 1:**
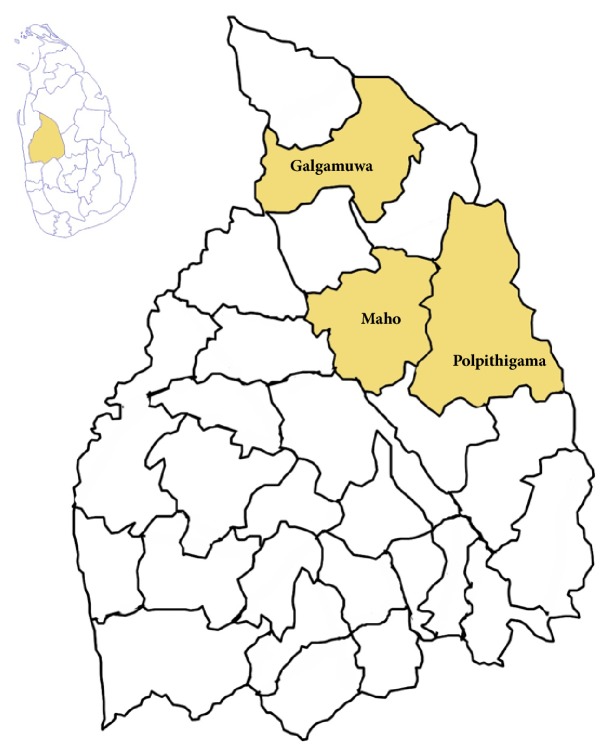
Study areas in Kurunegala District, Sri Lanka.

The District of Kurunegala has 26 Medical Officer of Health (MOH) areas. The prevalence of leishmaniasis in all MOH areas is not uniform in the district. The disease prevalence is considerably higher in seven MOH areas. Polpithigama, Maho, and Galgamuwa MOH areas are also in the list of high disease occurrence. Therefore, Polpithigama, Maho, and Galgamuwa MOH areas were selected for the present study (Figure 1).

### 2.3. Leishmaniasis Case Detection in Sri Lanka

The detection of the disease is done only through patients seeking medical attention for their symptoms. No program is conducted to detect patients at early stages of infection [7, 15]. The case detection is mainly achieved through microscopic observations of GIEMSA stained smears, while advanced techniques such as Polymerase Chain Reaction (PCR), Enzyme-Linked Immunosorbent Assay (ELISA), and other molecular diagnostic tests are available at very limited laboratories [9, 15].

### 2.4. Data Collection

Leishmaniasis patients who reside in Polpithigama, Galgamuwa, and Maho MOH areas and were notified of relevant MOH office during 2013- 2016 were obtained from the MOH office. Households of patients were visited and a pretested structured questionnaire was administered to the patients. If the patient is below 18 years, the parent or guardian was interviewed. In absence of the patient at the time of interview, the head of household or any responsible adult above 18 years nominated by the family was taken for the study as the most knowledgeable person.

In the collection of information on the source of income of patients, the sources were categorized as agricultural practices, nonagricultural practices, and monthly wages. Those who are involved in agriculture related activities such as chena and rice cultivation in their own land were considered under the agricultural practices category. Those who are involved in all other types of labour including masons, carpenters, and barbers were considered to be under the nonagricultural labour group. Those who were having government or private sector jobs with a salary at the end of the month were considered to be under the monthly wages category.

### 2.5. Data Tabulation

The questionnaire consisted of information on treatment seeking behaviour (health providers visited and mode of travel), direct and indirect costs associated with the health seeking behaviour and treatment. Direct medical and nonmedical costs of information were collected for each health seeking visit. All expenditures were assessed in Sri Lankan Rupees (Rs.) and converted to United States Dollars (USD) considering the average value of currency rate at the 31^st^ of December in each year from 2013 to 2016 according to the information available at the Central Bank of Sri Lanka (1 USD = Rs. 138.03).

#### 2.5.1. Medical Expenditure

These included all out of pocket expenditures by the household on consultation. In addition, the approximate costs of treatment and laboratory tests which are offered free of charge by the government hospitals/institutes were also considered under medical cost category.

#### 2.5.2. Direct Nonmedical Expenditure

Direct nonmedical costs comprise expenditure for transport, food costs, and other daily expenditures (if any) for the patient and accompanying family members.

#### 2.5.3. Indirect Expenditure

The indirect cost of an episode represented the loss of productivity within the household due to illness and was estimated using the human capital approach. The loss of productivity was estimated in terms of the loss of earnings of the patient and household members caring for the patient. For patients and attendants, the daily wage rate was estimated and multiplied by the number of work days lost to obtain the indirect cost of an episode. Here, those who depend on monthly wages were excluded when estimating the loss to household since there is no reduction of income due to the loss of a productive day. But all the patients were considered when estimating total economic loss at household level.

#### 2.5.4. Monthly and Annual Income of the Households

Monthly wage rate was noted by asking a series of questions on the monthly monetary income.

For patients and attendants reporting farming as their main source of income, the survey collected data on the yearly production of each produce which was then valued with local buying prices as expressed by participants and divided by the number of agriculturally active household members.

Total household income was also estimated as the sum of monthly cash income from daily labour for each economically active household member, income from agriculture, income from sales of animals /animal products (e.g., milk), and remittances from family members.

### 2.6. Data Analysis

The collected data were entered into Microsoft Excel Spreadsheets and analyzed using MINITAB software package (Version 17.0). The results were presented by descriptive statistics showing proportions, means, and standard deviations. The medians and interquartile ranges (25^th^ and 75^th^ percentiles) were also presented due to skewed distribution pattern in the expenditure data. The expenditures of households were defined as catastrophic, if they exceeded 10% of annual household income [4, 16].

## 3. Results

### 3.1. Sociodemographic and Epidemiological Aspect

A total of 121 patients were identified as CL patients recorded from three MOH areas from 2013 to 2016. Polpithigama MOH area was recorded to have the highest CL cases (n= 60), followed by Galgamuwa (n= 31), and Maho (n =30). Of the total recorded cases 70 households were accepted to be interviewed. Some households were not retrieved due to inaccuracy of the contact information provided to the MOH office and absence of patients at the named location.

The majority of the patients were males (n=36, 51%) and 83.4% (n= 30) of them were the chief occupants in the household. The females represented 49% (n=34) of the total patients interviewed. The median age of patients was 40 years (IQR= 29 – 53), while most patients were 25-50 years of age (n=38, 54%), followed by 50-75 years (n=14, 20%) and < 25 (n=11, 16%). Only 7 (10%) patients were > 75 years of age. The median number of members in a patient household of the current study was 4 (IQR=3-5).

Out of 70 patients, 57% (n=40) were economically active at the time of illness. Of them 65% (n=26) of the individuals who were identified as patients were the only persons contributing to the household income. The main source of income for the majority of patients was agricultural practices (n=26, 37%), followed by monthly wages (n=22, 31.5%), and other nonagricultural labor (n=22, 31.5%). The median monthly income of a patient was 22.56 USD (Rs. 3,114) (IQR= 15.24 – 35.80 USD), while the median monthly income of the household was 90.23 USD (Rs. 12,455) (IQR = 62.01 – 130.35 USD). Median annual per capita income of the study population (n=70) was 270.70 USD (Rs. 37,365) (IQR = 183.02 – 429.65 USD).

**Figure 2:**
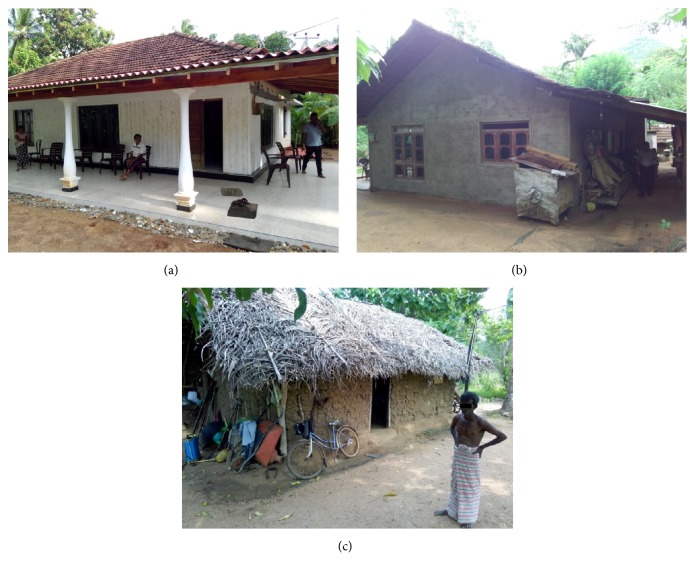
Examples of house types of patients. (a) Good (b) Moderate (c) Poor.

The houses having plastered cement walls with tiled or asbestos roofs were categorized as “Good” (Figure 2(a)), while unplastered brick walls with tiled or asbestos roofs were considered “Moderate” (Figure 2(b)). All other types were grouped as “Poor” houses (Figure 2(c)). Of these three categories, the “Moderate” house type was predominant (n=36, 53%) followed by good (n=24, 34%) and poor (n=9, 13%).

### 3.2. Health Care Receiving Information of the Patients

A median of 2 (IQR = 1.75 - 2) health care providers has been visited by patients for consultation, diagnosis, and treatments. The majority (73%) of patients had visited rural public hospitals, while others have directly gone to main public hospitals (n=17, 24%) or to a private clinic (Table 1). However, it is vital to emphasize that all patients had been diagnosed and treated by public hospitals, which the Sri Lankan Government maintains as a free service. Those who have visited private clinics as their first health seeking behaviour have spent an additional amount of money for consultation and medicine.

### 3.3. Diagnostic and Treatment Procedures

All patients have been diagnosed through both direct smear and skin biopsy evaluation at a public hospital having a dermatology clinic. Most of the patients were advised to use an antifungal cream, which had been also given by the hospital free of charge prior to the laboratory confirmation for CL. After diagnosis, majority of patients (n=67, 96%) have received treatment as cryotherapy using liquid nitrogen followed by an intralesional administration of Sodium Stibogluconate (SSG) at every treatment episode. Only 4% (n=3) had received treatment only with cryotherapy based on the diameter and site of the lesion. All patients have received treatments as outward patients. A median of 7 treatment episodes (IQR = 6-8) was received by a single patient. The minimum and maximum treatment episodes received by a patient were observed as 5 and 18, respectively. The highest number of 18 treatment episodes had been received by a 10-year-old child who had a recurrent lesion after first treatment.

### 3.4. Expenditure from Household Perspective

#### 3.4.1. Direct Costs

All the patients were diagnosed and treated at public hospitals, despite what they first visited. Therefore, costs for private clinics were limited only to consultation fee and the cost for initial medicines given to them. The median of direct expenditures by a patient was 58.32 USD (Rs. 8,050) (IQR = 49.48 – 70.63 USD), while 99.7% of the expenditures were denoted as nonmedical costs since all treatments have been received from public hospitals free of charge (Table 2). Remaining 0.3% was the cost borne for first treatment in private clinics prior to diagnosis of CL, which can be considered a negligible amount when compared to other nonmedical costs.

#### 3.4.2. Indirect Costs

Cutaneous leishmaniasis does not cause a significant morbidity. However, due to the requirement of multiple treatment episodes, the patients should travel a long distance to government public hospitals having dermatology clinics in order to receive treatments which in turn results in loss of productive days of both patients and the members who accompany them. Many study participants have indicated that the present illness had a considerable impact on their routine activities and resulted in loss of income to the household, either wage loss to both patients and accompanying person by the means of losses in agricultural output or other earnings. It is important to highlight that the psychological morbidities due to the presence of illness cannot be converted into a monetary value, which may have a direct influence on productivity.

Only 31 (44%) patients have been accompanied by someone to visit treatment facility. Out of those attendants 18 (26%) were economically active. From this group, the majority (n=13) were depending on daily wages and thus have an economic loss to their household. Total median indirect cost from household perspective at the end of the treatment was 20.67 USD (Rs. 2,853) (IQR = 0 – 32.05 USD) (Table 2). However, actual total median indirect cost to households at the completion of treatment was 13.37 USD (Rs. 1,846) (IQR = 0 – 26.74 USD) since there was no loss of income due to the loss of one productive day for those who depend on a monthly wage.

Total median costs including both direct and indirect costs were 78.47 USD (Rs. 10,831) (IQR = 67.20 – 101.85 USD), while total median economic loss to a household was 71.92 USD (Rs. 9,927) (IQR= 58.22 – 88.07 USD). The mean economic loss to households at completion of treatment was 76.59 USD (Rs. 10,572), which is about 5.4% of the annual household income and 20.9% of the mean annual per capita income of the study population.

### 3.5. Costs for Treatments from the Provider Perspective

The costs from provider perspective include expenditure for drugs, treatments, and laboratory investigations. The average diagnosis cost for a single patient was 2.32 USD (Rs. 320) for both direct smear and skin biopsy according to the information received from Government Public Hospitals having the treatment facility and government institutes offering diagnostic services. A single treatment episode is comprised of the application of liquid nitrogen onto the lesion from liquid nitrogen through a cryogun followed by an intralesional injection of Sodium Stibogluconate (SSG).

According to the information obtained from health care providers, the minimum expenditure for a single treatment episode was 3.83 USD (Rs. 528) (3.09 USD for SSG and 0.17 USD for cryotherapy). At the completion of all the treatment episodes, the median cost for SSG was 25.36 USD (Rs. 3,500) (IQR = 21.74 – 28.99 USD), while the median cost for cryotherapy is denoted as 1.42 USD (Rs. 196) (IQR = 1.22 -1.62). Thereby the total median cost from provider perspective at the end of all treatments accounted for 26.78 USD (Rs. 3,696) (IQR =22.91 –30.55 USD) for those who obtained both cryotherapy and SSG treatments (n= 67). Average cost for the patients who acquired only cryotherapy was approximately 2.32 USD (Rs. 320) per patient after all treatment episodes.

## 4. Discussion

This is the first study to provide information on estimating the cost and economic burden of cutaneous leishmaniasis in Sri Lanka. The study emphasizes that the cost of CL for patients and their family members was not high due to free provision of drugs and diagnosis by the Sri Lankan Government. It was observed that people rarely visit private health care providers in these areas. Some studies, conducted in Nepal, have indicated that the main reason for selecting first provider was due to close proximity and perceived reputation of the heath provider [4]. The selection of private health care provider by some patients in the present study was also noted as mainly due to closer proximity to their living places, thereby the convenience in reaching the place easily without spending more time and cost. It is interesting to note that no person had visited traditional healers as their health provider for the present illness even though some studies highlighted that more than 25% of the infected patients had selected the traditional healers [4, 17].

The majority of patients had first visited a rural public hospital located in the closest proximity to the house, and, based on medical examination, they have been referred to a main hospital for proper diagnosis and treatment, which conducts a dermatology clinic. These hospitals which provide treatment are not available at rural settings. Therefore, more than 59% of the study population happened to travel >100 km in order to receive treatment from the hospital and return. The vast majority of the patients (94%) had used public transport (bus) as the mode of transport. This is relatively cheaper rather than using private transport to travel this distance. However, the frequency of bus services in these rural settings is a limiting factor. Therefore, the patients may have to spend the whole day for treatment since it is more time consuming. On the other hand, there may be considerable rush at the dermatology clinics which usually operate one day per week. Since there are many referrals from different rural hospitals as this is the only focal place with treatment facilities and expertise, the whole day may be spent for this event. As many of the patients have spent more than 10 days to complete the treatment procedure, the present illness may cause the loss of productive hours of patients and the accompanying person. This may be one of the main reasons to increase both direct and indirect costs since it increases the costs for travelling and food and also results in the loss of a complete productive day. If the patient is a school student, the loss of school hours allocated for learning activities cannot be converted into a monetary value.

Importantly, a better health is central to human happiness and well-being. It also makes an important contribution to economic progress, as healthy populations live longer and are more productive [18]. Other neglected tropical diseases with prominent cutaneous manifestations like CL are socially damaging and deeply stigmatizing [19]. However, social stigma in leishmaniasis has also been shown to reinforce poverty in affected individuals and thus is of great concern [2]. Psychological investigations have shown that depression and anxiety are significantly higher in leishmaniasis patients [20, 21].

Many infectious vector-borne diseases can cause psychiatric and serious psychological conditions like phobias and posttraumatic stress disorders [22]. Therefore, the quality of life of patients depreciates considerably at acute phase and even after recovery [20, 23]. Therefore, it is also important to pay attention to postdisease psychological effects, in the case of CL, especially, which does not cause death or a significant morbidity but leaves a lifelong scar in exposed parts of the body which affects the normal life style of the patients even when they are completely healed. However, the economic burden associated with psychological morbidities due to CL cannot be estimated accurately.

Leishmaniasis is a relatively newly established disease in Sri Lanka. The first record was found in 1990s as an imported case [24]. The evidence of local transmission was detected in 1992 [25]. After that, several cases were recorded until 2008 when the disease was identified as a significant health problem in the country and a national action plan has been developed for the control of leishmaniasis [26]. Currently, Sri Lanka is considered one of the endemic countries for cutaneous leishmaniasis [15].

However, still there is no systematized plan for the identification of patients and vector control in disease endemic areas. Since majority of patients recorded from the country represent middle and low income communities in Sri Lanka, there are direct and indirect economic burdens on the patient households thereby depreciating their living standards and economic condition. Unfortunately, there are no published studies available in Sri Lanka in order to assess the economic consequences associated with leishmaniasis infection.

In Sri Lanka, the detection of the leishmaniasis disease is passive and only the patients seeking medical attention for their symptoms are screened for the presence of parasites [7, 15]. The patients in the present study attempted visiting a health care provider when there is a progressing lesion based on the locally acquired knowledge from the past CL infected patients or information and admonishes received from local community.

It is considered that, if an expenditure exceeding 10% of annual household income is catastrophic, this means that it drives households into destitution [16, 27]. The infected people in the present study represented a low income population and, thus, do not have the ability to pay for private health care. The current study showed that the economic impact is not catastrophic as the cost is only 5.4% of the mean annual household income. However, it is pretty much higher as a percentage of the mean annual per capita income which is 20.9%. This may be because of the fact that only one member of the family provides an income to the household, while others are dependents, which resulted in a lower per capita income. Therefore, the illness may cause a considerable impact on the economy of households.

When considering the economic loss from the provider's perspective, patients have undergone a diagnostic screening only once. Thus, the average cost was limited to only 2.32 USD (Rs. 320), which may be a negligible cost when compared to the other expenditures. However, there is a variation in treatment patterns and associated treatment expenses. The expenditure was more than 12 times higher for the patients who have been administered both SSG and cryotherapy treatments by the health provider (i.e., government public hospital on free of charge service) than those who received only cryotherapy. Therefore, cryotherapy appears to be an effective alternative to intralesion administration of SSG considering the significant side effects of pentavalent antimonial drugs [28, 29]. Anyhow, the cryotherapy alone could be used only when the lesion is smaller (around 1 cm or less) due to the lower compliancy rate around 40% [30].

The current study had several limitations such as the income and other cost information was obtained by an interviewer administered questionnaire. It was assumed that all the information provided by participants is accurate and there is no bias regarding the answers provided by them. On the other hand, the laborer costs and the costs for initial medicines provided to patients prior to diagnosis of CL were considered negligible with respect to other major expenditures. Conversely, seasonal variation in the economic loss was not considered. In addition, the patients were selected from the medical records archived at MOH offices. The records kept by the MOH offices were obtained through passive surveillance from cases detected and treated by public health facilities. Private for profit providers may not report patients treated at their facilities. Therefore, the actual case load may be underestimated.

Despite these limitations, the findings of the present study were still representative of a large proportion of the CL population in selected study area in Sri Lanka. Nonetheless, efforts should be made in the future to control this disease. More realistic and feasible approaches consist of reducing indirect costs by establishing treatment and diagnostic facilities at rural hospitals at disease endemic areas and correctly referring patients to nearby health centers and clinics could significantly reduce delays in diagnosis and treatment and the associated costs to rural households. This approach may help reduce the expenditure to patients at least by 40-50% since the majority of them have spent 58.32 USD (Rs. 8,050) for transportation and food during the treatment visits. Further, immediate actions are essential in order to increase the scientific knowledge about the disease and achieve higher effectiveness in the patient management. Control programmes must be accomplished through increased awareness of the disease among the general public and through active participation of local communities in control activities. It is therefore high time for health authorities to assess the need for the establishment of a Leishmaniasis Control Unit to conduct such activities in a systematized manner.

## 5. Conclusions

The economic impact of cutaneous leishmaniasis infection is not catastrophic when using mean annual household income as a proxy for the economic impact. However, the infection may have significant economic impacts on households when considering the mean economic loss to household as a percentage of the mean annual per capita income of the population. Cost per patient from the provider's perspective is relatively lower when compared to household level expenditures per patient. Cost is much lower when using cryotherapy alone than with intralesional SSG treatment, but it cannot be recommended due to lower compliancy rate and ineffectiveness against larger lesions.

## Figures and Tables

**Table 1:** Health care seeking information of cutaneous leishmaniasis patients.

**Variable**	**Number of patients (**%**)**
**Type of health care provider first visited**	
Private clinic	2 (3%)
Rural public hospitals	51 (73%)
Public hospital	17 (24%)
**Number of health care providers visited**	
1	17 (24%)
2	53 (76%)
**Visits till diagnosis for CL**	
1	0 (-)
2	22 (31%)
3	48 (69%)
**Mode of transportation to first health care provider**	
Own motor vehicle	4 (6%)
Bus	66 (94%)
**Mode of transportation to treatment facility**	
Own motor vehicle	3 (4%)
Bus	67 (96%)
**One way distance to the treatment facility from home (km)**	
21-30	0 (-)
31-40	2 (3%)
41-50	27 (38%)
51-60	23 (33%)
61-70	4 (6%)
71-80	4 (6%)
80 <	10 (14%)
**Number of productive days lost due to infection at the end of treatments**	
7	1 (1.4 %)
8	9 (12.9%)
9	13 (18.6%)
10	25 (35.7%)
11	16 (22.9)
12	2 (2.9%)
13	1 (1.4 %)
16	1 (1.4 %)
18	1 (1.4 %)
21	1 (1.4 %)
**Number of attending family members who lost productive days because of the patient**	
0	39 (56%)
1	31 (44%)

**Table 2:** Direct and indirect household costs associated with diagnosis and treatment.

**Type of the cost**	**Costs (USD)**			
	**Mean**	**SD**	**Median**	**IQR (25-75)**
**Direct nonmedical cost**				
Transportation	22.19	8.64	20.57	15.94 - 25.65
Food	38.73	14.66	36.22	31.15 – 47.82
Total direct nonmedical cost	60.92	17.99	58.32	49.48 – 70.64
**Indirect cost**				
Loss of income of patients	19.00	222.41	16.54	0 – 23.07
Loss of income of accompanying family members	6.16	111.22	0	0 - 11.37
Total indirect cost	25.63	222.89	20.66	0 – 32.06

## Data Availability

The data used to support the findings of this study are available from the corresponding author upon request.
